# Characterizing mothers and children at risk of being under-immunized in India: A latent class analysis approach

**DOI:** 10.1016/j.ijid.2020.08.056

**Published:** 2020-11

**Authors:** Taylor A. Holroyd, Brian Wahl, Madhu Gupta, Molly Sauer, Madeleine Blunt, Amelia K. Gerste, Daniel J. Erchick, Mathuram Santosham, Rupali J. Limaye

**Affiliations:** a Department of International Health, Johns Hopkins Bloomberg School of Public Health, Baltimore, Maryland, USA; b International Vaccine Access Center, Johns Hopkins Bloomberg School of Public Health, Baltimore, Maryland, USA; c Department of Community Medicine and School of Public Health, Postgraduate Institute of Medical Education and Research, Chandigarh, India; d Department of Epidemiology, Johns Hopkins Bloomberg School of Public Health, Baltimore, Maryland, USA; e Department of Health, Behavior and Society, Johns Hopkins Bloomberg School of Public Health, Baltimore, Maryland, USA

**Keywords:** Immunization, Inequalities, India, Child health, Latent class analysis

## Abstract

**Objectives:**

While India has made substantial progress in introducing new vaccines and scaling up immunization coverage, inequities persist sub-nationally. This study was performed to investigate the risk of under-immunization based on class membership and to identify heterogeneous classes based on sociodemographic characteristics in pediatric and maternal populations in India through latent class analysis.

**Methods:**

Data from the most recent National Family Health Survey conducted in 2015–2016 were used. Latent class analysis was used to model immunization coverage in children aged 12–23 months and mothers, and to identify subgroups to characterize those at risk of not being immunized.

**Results:**

Patterns of sociodemographic characteristics were found to contribute to non-immunization or under-immunization among pediatric and maternal populations in India. Individuals who fit into one of three categories were identified in both populations: those at high, medium, and lower risk of not being immunized. Lower socioeconomic status, lack of antenatal care, and lower maternal education put individuals at higher risk of not being immunized with routine childhood vaccines and maternal tetanus toxoid.

**Conclusions:**

Predisposing risk factors can persistently impact immunization status despite improvements in immunization access in India. Tailored programmatic interventions should be developed to improve immunization coverage among those children and mothers who are at highest risk of being under-immunized or not immunized.

## Highlights

• Immunization inequities persist in India despite substantial gains.
• Sociodemographic characteristics contribute to non-immunization or under-immunization.
• Tailored interventions can improve immunization coverage for those at high risk.

## Introduction

Vaccines have played a pivotal role in improving child health in India. In recent years, India has achieved several significant immunization-related landmarks, including being certified polio-free in 2014 and eliminating maternal and neonatal tetanus in 2015 (Ministry of Health & Family Welfare, 2014; World Health Organization, 2016). India first introduced routine immunization in 1978 as the Expanded Programme on Immunization (EPI). This program was later expanded in 1985 as the Universal Immunization Programme (UIP), and India’s immunization program is now the largest in the world in terms of geographic and population coverage. The UIP currently provides vaccines that provide protection from 12 vaccine-preventable diseases, and all vaccines are available free of cost to India’s annual birth cohort (Travasso, 2015). Long-included vaccines include bacillus Calmette–Guérin (BCG) vaccine, pentavalent vaccine protecting against diphtheria, tetanus, pertussis, *Haemophilus influenzae* type b, and hepatitis B, oral polio vaccine, and inactivated polio vaccine. Since 2016, the government has also included rotavirus vaccine, measles–rubella vaccine, and pneumococcal conjugate vaccine within the UIP (International Institute for Population Sciences (IIPS) and ICF, 2017). However, in 2015–2016, the average national full immunization coverage was 62%, with only a few states achieving and sustaining coverage of more than 85% (International Institute for Population Sciences (IIPS) and ICF, 2017).

Maternal immunizations provide crucial health benefits and protection against vaccine-preventable infections for both mothers and their newborns. The only vaccine included specifically for pregnant women in India’s national immunization schedule prior to 2018 was tetanus toxoid (TT) vaccine. As of 2018, TT has been replaced by diphtheria–tetanus vaccine (DT) to prevent maternal and neonatal infections during prenatal care (National Health Mission, 2018). The UIP recommended that pregnant women who had not previously been vaccinated with TT vaccine or whose immunization status was unknown receive two doses of TT vaccine: the first dose given in early pregnancy, followed by the second 1 month later. A booster dose was recommended for pregnant women who had received two TT doses in a pregnancy within the past 3 years (Ministry of Health and Family Welfare, 2008). Maternal and neonatal tetanus are among the leading life-threatening consequences of unhygienic umbilical cord care practices and deliveries, exposing inequities within countries in access to immunization and other antenatal and neonatal care services (World Health Organization, 2020). India achieved elimination of tetanus in April 2015 and relies on sustained efforts focused on the routine immunization of children, mothers, and women of reproductive age and the promotion of hygienic deliveries and cord care practices to maintain this status (World Health Organization, 2016).

While India has made substantial gains in introducing new vaccines and scaling up immunization coverage, inequities persist sub-nationally—both between and within states. National full immunization coverage increased from 43.5% in 2005–2006 to 62% in 2015–2016, an improvement of more than 42% (International Institute for Population Sciences (IIPS) and ICF, 2007, International Institute for Population Sciences (IIPS) and ICF, 2017). However, the coverage gap among states/union territories narrowed only marginally during that period. In 2005–2006, coverage ranged from 21% in Nagaland to 80.9% in Tamil Nadu; in 2015–2016, coverage ranged from 35.4% in Nagaland to 91.2% in Puducherry (International Institute for Population Sciences (IIPS) and ICF, 2007, International Institute for Population Sciences (IIPS) and ICF, 2017). Northeastern states were disproportionately among those with the highest proportions of unvaccinated children 12–23 months of age in both survey years; in 2015–2016, one in every five children in Mizoram was unvaccinated. Furthermore, studies have noted historic gender inequities in immunization, with girls less likely to receive routine vaccinations than boys, although progress has been made in closing this gender gap (Corsi et al., 2009). Recognizing continued sub-national coverage gaps, the Government of India launched Mission Indradhanush in December 2014 to conduct targeted immunization drives in districts with low full immunization coverage (Gurnani et al., 2018). The initiative expanded through subsequent phases, aiming to reach 90% full immunization and close sub-national coverage gaps.

Globally, a number of risk factors have been identified that contribute to non-immunization or under-immunization with routinely recommended vaccines among both children and women. Lower socioeconomic status, living in rural settings, and poor access to healthcare facilities are widely associated with children not being fully immunized on time (Cao et al., 2018, Crocker-Buque et al., 2017, Hu et al., 2017, Maekawa et al., 2007, Noh et al., 2018). Among women of reproductive age and pregnant women, poor access to antenatal care, limited maternal education, and lack of skilled attendance at delivery have been associated with decreased odds of TT immunization (Dempsey et al., 2016, Gupta and Keyl, 1998, Vojtek et al., 2018). In addition to suboptimal vaccination and an increased risk of infectious disease, these risk factors also contribute to a wide range of health issues that are particularly concerning in populations of lower socioeconomic status.

Mitigating inequities in immunization is essential to increase vaccine acceptance and subsequent coverage, and relies on the ability of program managers to be aware of and understand risk factors that can inform intervention efforts related to programs as well as policies (Egondi et al., 2015, Pande and Yazbeck, 2003). The stark inequities in immunization in India further indicate a need to assess the sociodemographic factors that contribute to immunization status. Understanding these factors can have important implications for vaccine policy and programming in terms of identifying predictive risk factors and developing tailored interventions to ensure vaccine access and uptake. An examination of the interplay of multiple risk factors can provide a more thorough understanding of influences on vaccine decision-making than that provided by studying risk factors in isolation (Lanza and Rhoades, 2013). Finite mixture models provide a statistically sophisticated framework for identifying subgroups of individuals who are not directly observable. Specifically, latent class analysis (LCA) is a statistical method for identifying underlying groups of similar individuals or units from a heterogeneous sample.

Despite significant improvements in vaccine access and coverage, pediatric and maternal vaccine coverage in India remains suboptimal, with notable inequities between states and populations. Identifying subgroups at risk of non-immunization and under-immunization is crucial for vaccine policy and programming. The aims of this study were to identify heterogeneous classes based on sociodemographic characteristics in pediatric and maternal populations in India through LCA, and to investigate the risk of not being fully immunized based on sociodemographic characteristics and class membership.

## Methods

### Data source

Data from the most recent National Family Health Survey (NFHS) conducted in 2015–2016 (NFHS-4) were used (International Institute for Population Sciences (IIPS) and ICF, 2017). This is a nationally representative survey that collected data from a two-stage stratified sample of 601 509 households in 36 states and union territories in India (International Institute for Population Sciences (IIPS) and ICF, 2017).

### Variables

Full immunization status was assessed in two different populations drawn from the national survey data: children aged 12–23 months and mothers. Among the pediatric population, children were counted as being fully vaccinated if they had received three doses of diphtheria–tetanus–pertussis-containing (DTP) vaccine, three doses of polio vaccine, one dose of BCG vaccine, and one dose of measles-containing vaccine. They were counted as not fully vaccinated if they had not received one or more of these vaccines. Among the maternal population, mothers were counted as being fully vaccinated if they had received TT vaccine before their most recent child’s birth. For both populations, immunization status was assessed by immunization cards or maternal recall.

A number of factors that may be associated with full immunization status for both children and mothers were identified based on previous literature (Cao et al., 2018, Crocker-Buque et al., 2017, Dempsey et al., 2016, Gupta and Keyl, 1998, Hu et al., 2017, Maekawa et al., 2007, Noh et al., 2018, Vojtek et al., 2018). Factors associated with child immunization included gender, birth order, religion, caste, socioeconomic status, maternal education, ease of reaching a healthcare facility, geographic setting, number of antenatal care visits, and skilled attendant at delivery. Factors associated with maternal immunization included maternal age, religion, caste, socioeconomic status, maternal education, ease of reaching a healthcare facility, geographic setting, number of antenatal care visits, and skilled attendant at delivery. Multicollinearity of the included variables was assessed using the *vif* command in Stata in order to ensure local independence of the included variables.

### Data analysis

An inclusive approach to LCA was used to model immunization coverage and identify subgroups that vary in immunization status to characterize those at risk of not being immunized in the pediatric and maternal populations. To identify the optimal number of classes for each population, the Akaike information criterion (AIC), Bayesian information criterion (BIC), and sample-size-adjusted Bayesian information criterion (SSA-BIC) were examined, with lower values of AIC, BIC, and SSA-BIC indicating a better fit. The Lo–Mendell–Rubin likelihood ratio test (LMR-LRT) was used to compare successive class models, with significant LMR-LRT values indicating a substantial improvement when comparing the k-class model to the (k – 1)-class model.

Regarding the examination of the predictors of class membership, the Pearson Chi-square test was used to examine statistically significant differences in sociodemographic characteristics between classes. Multinomial logistic regression was conducted to confirm which variables best differentiated between classes and predicted class membership.

The survey sampling design was taken into account in all analyses; the NFHS-4 collected data at both the district and state level with a two-stage stratified sample (International Institute for Population Sciences (IIPS) and ICF, 2017). The 2011 India census was used as the sampling frame for the NFHS-4, with villages in rural areas and census enumeration blocks in urban areas used as the primary sampling units (International Institute for Population Sciences (IIPS) and ICF, 2017). LCA was performed using Mplus version 8 (Mplus, Los Angeles, CA, USA). All other data cleaning and analyses were done using Stata version 15 (StataCorp, College Station, TX, USA).

## Results

### Sample description

Among the pediatric population (*n* = 49 284), the average age of participants was 17.4 months (standard deviation (SD) 3.4 months) and 52% were male. Among the maternal population (*n* = 259 627), the average age of participants was 27.2 years (SD 5.2 years), and they had given birth to a mean of 2.6 children (SD 1.3). Table 1 demonstrates bivariate associations between identified risk factors and immunization status for both the maternal and child populations.

**Table 1:** Likelihood of respondents being fully immunized associated with sociodemographic risk factors.

Sociodemographic variable	Unadjusted OR (95% CI)	
	Pediatric population (*n* = 49 284)	Maternal population (*n* = 259 627)
Religion		
Hindu	1.00	1.00
Muslim	0.72 (0.69–0.76)	0.67 (0.64–0.70)
Other	0.82 (0.77–0.86)	0.49 (0.47–0.51)
Geographic setting		
Urban	1.00	1.00
Rural	0.81 (0.78–0.85)	0.67 (0.64–0.70)
Maternal education		
None	1.00	1.00
Incomplete primary	1.35 (1.25–1.47)	1.44 (1.35–1.53)
Complete primary	1.39 (1.30–1.50)	1.83 (1.72–1.95)
Incomplete secondary	1.83 (1.75–1.92)	2.29 (2.20–2.37)
Complete secondary	2.20 (2.05–2.37)	2.86 (2.66–3.06)
Higher	2.59 (2.42–2.78)	3.27 (3.05–3.50)
Socioeconomic status		
Poorest	1.00	1.00
Poorer	1.36 (1.30–1.43)	1.40 (1.34–1.46)
Middle	1.78 (1.68–1.87)	1.94 (1.85–2.03)
Richer	2.04 (1.93–2.16)	2.27 (2.16–2.39)
Richest	2.55 (2.39–2.72)	3.46 (3.25–3.68)
Ease reaching health facility		
No problem	1.00	1.00
Not a significant problem	0.80 (0.76–0.84)	0.77 (0.74–0.80)
Significant problem	0.62 (0.59–0.65)	0.47 (0.45–0.49)
Antenatal care		
No visits	1.00	1.00
Insufficient <4 visits	2.16 (2.05–2.28)	6.57 (6.30–6.85)
Sufficient ≥4 visits	3.55 (3.37–3.74)	7.89 (7.59–8.21)
Caste		
General	1.00	1.00
SC/ST	0.75 (0.72–0.80)	0.58 (0.56–0.61)
OBC	0.92 (0.87–0.97)	0.87 (0.83–0.92)
Skilled attendant at delivery		
No	1.00	1.00
Yes	1.76 (1.69–1.83)	2.48 (2.40–2.56)
Birth order		
1^st^	1.00	NA
2^nd^	0.85 (0.81–0.89)	
3^rd^	0.68 (0.65–0.72)	
4^th^	0.57 (0.53–0.61)	
5^th^ or greater	0.43 (0.40–0.46)	
Gender		
Male	1.00	NA
Female	1.00 (0.96–1.04)	

OR, odds ratio; CI, confidence interval; SC/ST, Scheduled Castes/Scheduled Tribes; OBC, Other Backward Class; NA, not applicable.

### Pediatric population

For the classes focused on pediatric immunization, latent class models with two to five classes showed distinct decreases in the AIC, BIC, and SSA-BIC values, with the lowest values for the five-class model (Table 2). Significant LMR-LRT values were observed for all models with more than one class. The LMR-LRT was found to overestimate the class number, so the five-class model was excluded. Comparing the higher-class models, the four-class model did not substantially improve upon the three-class model according to the latent class probabilities. However, the three-class model did improve upon the two-class model according to the latent class probabilities. As such, the three-class model was selected as the optimal solution based on fit statistics and parsimony, with class 1 (*n* = 15 259, 31%) labeled as higher risk, class 2 (*n* = 21 903, 44%) labeled as medium risk, and class 3 (*n* = 12 122, 25%) labeled as lower risk of not being fully immunized.

**Table 2:** Goodness-of-fit statistics for pediatric population models.

Model	Log-likelihood	AIC	BIC	SSA-BIC	LMR-LRT	LMR *p*-value
1 class	−511 843.8	1 023 741.6	1 023 979.3	1 023 893.5	NA	NA
2 class	−487 248.6	974 607.2	975 091.5	974 916.7	49 028.3	0.0000
3 class	−481 970.1	964 106.3	964 837.1	964 573.3	10 520.9	0.0000
4 class	−479 161.0	958 544.0	959 521.4	959 168.6	5599.8	0.0000
5 class	−477 376.0	955 030.0	956 253.9	955 812.2	3558.3	0.0000

AIC, Akaike information criterion; BIC, Bayesian information criterion; SSA-BIC, sample-size-adjusted BIC; LMR-LRT, Lo–Mendell–Rubin likelihood ratio test; NA, not applicable.

### Predictors of class membership: pediatric population

Table 3 shows sociodemographic variables for individuals in each of the three pediatric classes. Results of Pearson Chi-square tests indicated that there were significant differences among the subgroups in terms of religion, geographic setting, maternal education, socioeconomic status, ease of reaching a health facility, birth order, antenatal care, caste, and the presence of a skilled attendant at delivery. Specifically, lower socioeconomic status (compared to a higher socioeconomic status), residing in a rural setting (compared to an urban setting), lower maternal education (compared to higher levels of education), difficulty reaching a health facility, higher birth order, and less antenatal care were all associated with an increased risk of a child not being fully immunized with routine vaccines (*p* < 0.001).

**Table 3:** Sociodemographic and immunization-related characteristics across pediatric latent classes (*n* = 49 284).

Sociodemographic variable	Class 1 Higher risk (*n* = 15 259, 31%)	Class 2 Medium risk (*n* = 21 903, 44%)	Class 3 Lower risk (*n* = 12 122, 25%)	*p*-Value
Fully immunized	7342 (48.12)	14 193 (64.8)	8610 (71.0)	
Religion				<0.001
Hindu	10 813 (70.9)	16 413 (74.9)	8198 (67.6)	
Muslim	2861 (18.8)	2698 (12.3)	2330 (19.2)	
Other	1585 (10.4)	2792 (12.8)	1594 (13.2)	
Geographic setting				<0.001
Urban	872 (5.7)	3051 (13.9)	7242 (59.7)	
Rural	13 892 (91.0)	17 365 (79.3)	4158 (34.3)	
Not a de jure resident	495 (3.2)	1487 (6.8)	722 (5.96)	
Maternal education				<0.001
None	11 075 (72.6)	2431 (11.1)	546 (4.5)	
Incomplete primary	1457 (9.6)	1492 (6.8)	117 (1.0)	
Complete primary	1364 (8.9)	2154 (9.8)	439 (3.6)	
Incomplete secondary	1302 (8.5)	12 957 (59.2)	4443 (36.7)	
Complete secondary	61 (0.4)	1940 (8.9)	2355 (19.4)	
Higher	0 (0.0)	929 (4.2)	4222 (34.83)	
Socioeconomic status				<0.001
Poorest	10 229 (67.0)	2340 (10.7)	0 (0.0)	
Poorer	3816 (25.0)	7562 (34.5)	0 (0.0)	
Middle	946 (6.2)	8615 (39.3)	462 (3.8)	
Richer	205 (1.3)	3386 (15.46)	4857 (40.1)	
Richest	63 (0.4)	0 (0.0)	6803 (56.1)	
Ease reaching health facility				<0.001
No problem	2115 (13.9)	6069 (27.7)	7301 (60.2)	
Not a significant problem	4948 (32.4)	8401 (38.4)	3353 (27.7)	
Significant problem	8196 (53.7)	7433 (33.94)	1468 (12.1)	
Birth order				<0.001
1^st^	2482 (16.3)	9683 (44.2)	6037 (49.8)	
2^nd^	3488 (22.9)	7815 (35.7)	4362 (36.0)	
3^rd^	3525 (23.1)	3078 (14.1)	1238 (10.2)	
4^th^	2576 (16.9)	984 (4.5)	338 (2.8)	
5^th^	1507 (9.9)	260 (1.2)	89 (0.7)	
6^th^	823 (5.4)	83 (0.4)	31 (0.3)	
≥7^th^	858 (5.6)	0 (0.0)	27 (0.2)	
Gender				0.056
Male	7797 (51.1)	11 456 (52.3)	6327 (52.2)	
Female	7462 (48.9)	10 447 (47.7)	5795 (47.8)	
Antenatal care				<0.001
No visits	5673 (37.2)	1800 (8.2)	567 (4.7)	
Insufficient <4 visits	6396 (41.9)	7517 (34.3)	2428 (20.0)	
Sufficient ≥4 visits	2337 (15.3)	11 240 (51.3)	8559 (70.6)	
Not reported	853 (5.6)	1346 (6.2)	568 (4.7)	
Caste				<0.001
SC/ST	7625 (50.0)	9064 (41.4)	2433 (20.1)	
OBC	5755 (37.7)	8430 (38.5)	5137 (42.4)	
General	1275 (8.4)	3301 (15.1)	3996 (33.0)	
Skilled attendant at delivery				<0.001
No	8731 (57.2)	5337 (24.4)	1748 (14.4)	
Yes	6528 (42.8)	16 566 (75.6)	10 374 (85.6)	

SC/ST, Scheduled Castes/Scheduled Tribes; OBC, Other Backward Class.

### Maternal population

Examining classes related to maternal immunization, latent class models with two to five classes showed distinct decreases in the AIC, BIC, and SSA-BIC values, with the lowest values for the five-class model (Table 4). Significant LMR-LRT values were observed for the two, three, four, and five-class models. Comparing the remaining models, the three-class model was found to improve upon the two-class model according to the latent class probabilities. Thus, the three-class model was selected as the best fit for the maternal population based on fit statistics and parsimony, with class 1 (*n* = 83 610, 32%) labeled as higher risk, class 2 (*n* = 117 674, 45%) labeled as medium risk, and class 3 (*n* = 58 343, 23%) labeled as lower risk of not being fully immunized with TT vaccine.

**Table 4:** Goodness-of-fit statistics for maternal population models.

Model	Log-likelihood	AIC	BIC	SSA-BIC	LMR test	LMR *p*-value
1 class	−2 426 387.6	4 852 823.2	4 853 074.4	4 852 998.2	NA	NA
2 class	−2 308 847.0	4 617 791.9	4 618 304.8	4 618 149.1	234 329.5	0.0000
3 class	−2 279 578.9	4 559 305.7	4 560 080.3	4 559 845.1	58 349.0	0.0000
4 class	−2 267 602.7	4 535 403.5	4 536 439.7	4 536 125.1	23 872.5	0.0000
5 class	−2 260 199.7	4 520 647.5	4 521 945.4	4 521 551.3	14 758.7	0.0000

AIC, Akaike information criterion; BIC, Bayesian information criterion; SSA-BIC, sample-size-adjusted BIC; LMR-LRT, Lo–Mendell–Rubin likelihood ratio test; NA, not applicable.

### Predictors of class membership: maternal population

Table 5 shows sociodemographic variables for individuals in each of the three maternal classes. Results from Pearson Chi-square tests indicated that there were significant differences among the subgroups in terms of maternal age, religion, geographic setting, maternal education, socioeconomic status, ease of reaching a health facility, antenatal care, caste, and skilled attendant at delivery. Specifically, younger maternal age (mainly 20–25 years), lower socioeconomic status (compared to a higher socioeconomic status), residing in a rural setting (compared to an urban setting), lower maternal education, difficulty reaching a health facility, and less antenatal care were all associated with an increased risk of a woman not being fully immunized with TT vaccine (*p* < 0.001).

**Table 5:** Sociodemographic and immunization-related characteristics across maternal latent classes (*n* = 259 627).

Sociodemographic variable	Class 1 Higher risk (*n* = 83 610, 32%)	Class 2 Medium risk (*n* = 117 674, 45%)	Class 3 Lower risk (*n* = 58 343, 23%)	*p*-Value
Fully immunized	45 863 (54.9)	80 837 (68.7)	44 487 (76.3)	
Maternal age in years				<0.001
<20	1615 (1.9)	4709 (4.0)	375 (1.0)	
20–24	17 433 (20.9)	47 907 (40.7)	12 837 (22.0)	
25–29	29 528 (35.3)	44 890 (38.2)	24 978 (42.8)	
30–34	19 499 (23.3)	15 291 (13.0)	14 215 (24.4)	
>35	15 535 (18.6)	4877 (4.1)	5938 (10.2)	
Religion				<0.001
Hindu	60 951 (72.9)	87 162 (74.1)	39 460 (67.6)	
Muslim	14 157 (16.9)	15 909 (13.5)	10 884 (18.7)	
Other	8502 (10.2)	14 603 (12.4)	7999 (13.7)	
Geographic setting				<0.001
Urban	3474 (4.2)	18 571 (15.8)	36 080 (61.8)	
Rural	77 046 (92.2)	91 964 (78.2)	18 731 (32.1)	
Not a de jure resident	3090 (3.7)	7139 (6.1)	3532 (6.1)	
Maternal education				<0.001
None	62 220 (74.4)	15 821 (13.44)	3046 (5.2)	
Incomplete primary	7726 (9.2)	8613 (7.3)	601 (1.0)	
Complete primary	6585 (7.9)	12 369 (10.5)	2044 (3.5)	
Incomplete secondary	6903 (8.3)	66 652 (56.6)	21 813 (37.4)	
Complete secondary	132 (0.2)	9988 (8.5)	11 158 (19.1)	
Higher	44 (0.1)	4231 (3.6)	19 681 (33.7)	
Socioeconomic status				<0.001
Poorest	58 407 (69.9)	10 289 (8.7)	0 (0.0)	
Poorer	21 285 (25.5)	40 151 (34.1)	0 (0.0)	
Middle	3402 (4.1)	46 733 (39.7)	1627 (2.8)	
Richer	399 (0.5)	20 386 (17.3)	22 161 (38.0)	
Richest	117 (0.1)	115 (0.1)	34 555 (59.2)	
Ease reaching health facility				<0.001
No problem	11 278 (13.5)	33 617 (28.6)	34 880 (59.8)	
Not a significant problem	26 531 (31.7)	45 556 (38.7)	16 331 (28.0)	
Significant problem	45 801 (54.8)	38 501 (32.7)	7132 (12.2)	
Antenatal care				<0.001
No visits	23 666 (28.3)	7500 (6.4)	2476 (4.2)	
Insufficient <4 visits	24 074 (28.8)	31 616 (26.9)	10 274 (17.6)	
Sufficient ≥4 visits	9089 (10.9)	47 412 (40.3)	34 791 (59.6)	
Not reported	26 781 (32.0)	31 146 (26.5)	10 802 (18.5)	
Caste				<0.001
SC/ST	6385 (7.6)	19 200 (16.3)	19 434 (33.3)	
OBC	42 976 (51.4)	46 379 (39.4)	11 895 (20.4)	
General	30 937 (37.0)	46 319 (39.4)	24 530 (42.0)	
Not reported	3312 (4.0)	5776 (4.9)	2484 (4.3)	
Skilled attendant at delivery				<0.001
No	49 889 (59.7)	30 186 (25.7)	8442 (14.5)	
Yes	33 616 (40.2)	87 452 (74.3)	49 884 (85.5)	

SC/ST, Scheduled Castes/Scheduled Tribes; OBC, Other Backward Class.

### Multinomial logistic regression

Multinomial logistic regression analysis was conducted to compare the identified latent classes and examine which variables were the most important predictors of class membership. Maternal age, religion, geographic setting, maternal education, socioeconomic status, ease of reaching a health facility, antenatal care, caste, and skilled attendant at delivery were confirmed to be significant predictors of class membership for the maternal population. Religion, geographic setting, maternal education, socioeconomic status, ease of reaching a health facility, birth order, antenatal care, caste, and skilled attendant at delivery were confirmed to be significant predictors of class membership for the pediatric population; gender was again not a significant predictor of class membership. Of particular note, in both pediatric and maternal populations, individuals in the higher risk class were significantly more likely to have lower socioeconomic status, limited antenatal care, and less maternal education.

## Discussion

The results of this study demonstrate patterns of sociodemographic characteristics that contribute to non-immunization or under-immunization among pediatric and maternal populations in India. In both groups, three classes we identified and subsequently characterized as being at higher, medium, and lower risk of not being immunized, respectively. In particular, lower socioeconomic status, lack of antenatal care, and lower maternal education were identified as especially important factors that put individuals at higher risk of not being immunized with routine childhood vaccines or maternal TT. It appears that this type of analysis has not previously been applied to the most recent NFHS dataset, leaving a wide gap in the literature regarding whether the same risk factors still apply. These risk factors are consistent with those identified in studies that did not utilize LCA (Cao et al., 2018, Crocker-Buque et al., 2017, Hu et al., 2017, Maekawa et al., 2007, Noh et al., 2018). These findings have important policy and programming implications for the Indian immunization context. While it has long been understood that individuals of lower socioeconomic status are less likely to be immunized, the present study results will aid policymakers in more easily identifying high-risk populations and more effectively tailoring interventions to reach both mothers and children in India.

Notably, despite the elimination of maternal and neonatal tetanus in India in 2015 (World Health Organization, 2016), we observed wide variation in TT immunization status among mothers in the NFHS survey. The risk factors that contribute to under-vaccination are complex. Barriers to vaccine acceptance vary widely depending on context and target population. As certain risk factors may place groups of individuals at higher risk of under-vaccination, it may not always be possible or recommended to apply a ‘one-size-fits-all’ principle with regards to a vaccination program. Careful consideration of risk factors and interests is required to develop a more targeted approach, so that programs are tailored to specific immunization risk factors (Kimman et al., 2006). To improve efficiency in resource-constrained settings, vaccination strategies should be tailored in order to fit the needs of the people most at risk of not being immunized (Rots et al., 2010, Samad et al., 2006). For example, countries could consider developing vaccination programs that are tailored to individual risk factors like socioeconomic status, lower maternal education, and access to antenatal care. For this to be feasible, surveillance should prioritize monitoring of vaccination coverage of different risk groups. Tailoring interventions to specific risk factors can also enable policymakers to better address immunization inequalities between and within populations.

This study identified factors associated with childhood and maternal immunization status that operate on multiple levels of influence. Socio-ecological models have been used previously to describe how complex factors operate to determine health outcomes across individual, interpersonal, organizational, community, and societal/policy levels (Golden and Earp, 2012). Evidence suggests that interventions to improve vaccine acceptance, for example, should consider how barriers can be addressed through multi-component strategies that are tailored to specific populations and concerns (Jarrett et al., 2015). India’s Mission Indradhanush program, launched in 2014 to increase full immunization coverage of children to 90%, adopted aspects of this approach by specifically targeting underserved and hard-to-reach populations (Gurnani et al., 2018). While directly modifying most distal societal factors – such as socio-economic status, geographic setting, or maternal education – may be impractical for health programs on a broad scale, understanding how these factors affect low coverage can help policymakers and health officials tailor structural interventions to specific populations.

Evidence from India suggests that maternal health knowledge and communications skills are pathways through which educational interventions might positively impact immunization coverage (Vikram et al., 2012). Community-level factors and related interventions, such as those involving religious or traditional leaders, have yielded positive evidence of effects on vaccine uptake and confidence (Ansari et al., 2007, Jarrett et al., 2015, Nasiru et al., 2012). Organizational-level risk factors were also important predictors in the present analysis – particularly health systems factors, including antenatal care coverage, skilled attendance at delivery, and ease of reaching a health facility. Coverage of antenatal and perinatal services have increased dramatically in India in recent years, presenting an opportunity to encourage uptake of newborn and child health interventions and deliver health education at these earlier touchpoints in the continuum of care (International Institute for Population Sciences (IIPS) and ICF, 2017). Last, there is increasing recognition of the complexity and interconnectedness of the interpersonal and individual-level factors that most proximally determine the immunization-related knowledge, perceptions, and behaviors of primary caregivers and families (Gupta et al., 2017, MacDonald, 2015). These factors, which are not captured through standard survey methodologies, were not included in our analysis; however, their influence on immunization coverage, through the action of vaccine hesitancy, confidence, and demand, should not be overlooked, particularly as supply-side barriers are overcome.

There are several limitations related to a LCA approach. First, only observed indicators can be analyzed, particularly given the use of secondary data. Second, an a priori hypothesis regarding the number of classes and the characteristics of each class cannot be established. Third, this method assumes that full immunization coverage can be appropriately captured as a categorical variable. While the risk factors used in this analysis were grounded in existing literature, more indicators available from the NFHS data could be employed to identify additional predictors of a lack of immunization. However, this analysis also has several notable strengths. The very large sample size afforded by the NFHS data ensured that the LCA was sufficiently powered for class enumeration. The NFHS sample is also fairly heterogeneous in terms of sociodemographic characteristics, given it is intended to represent regional and national populations in India. The NFHS data could include multiple children from the same household, potentially creating observations that are not independent, but this is unlikely as there would be very few families with multiple children aged 12–23 months.

## Conclusions

This study provides strong evidence that children and mothers in India can be differentiated into classes that are at higher, medium, and lower risk of not being immunized. This highlights the predisposing risk factors that can persistently impact immunization status despite improvements in immunization availability and access in India. We observed different patterns of socioeconomic and demographic characteristics that are associated with varying immunization subgroups. In particular, we identified lower socioeconomic status, lack of antenatal care, and lower maternal education as especially important factors that put individuals at higher risk of not being fully immunized. The results can enable policymakers to better characterize and identify children and mothers in India who are less likely to be fully immunized. Tailored programmatic interventions can be developed to increase vaccine access in hard-to-reach populations and improve immunization coverage among those children and mothers who are at highest risk of not being immunized or being under-immunized.

## Funding source

This study was funded by the Bill & Melinda Gates Foundation. The funder played no role in the study design, in the collection, analysis and interpretation of the data, in the writing of the report, or in the decision to submit the article for publication.

## Ethical approval

Approval for this study was not required as it pertains to the analysis of secondary data.

## Conflict of interest

The authors declare that they have no known competing financial interests or personal relationships that could have appeared to influence the work reported in this article.

## References

[bib0005] Ansari M.A., Khan Z., Khan I.M. (2007). Reducing resistance against polio drops. J R Soc Promot Health.

[bib0010] Cao L., Zheng J.S., Cao L.S., Cui J., Duan M.J., Xiao Q.Y. (2018). Factors influencing the routine immunization status of children aged 2-3 years in China. PLoS One.

[bib0015] Corsi D.J., Bassani D.G., Kumar R., Awasthi S., Jotkar R., Kaur N. (2009). Gender inequity and age-appropriate immunization coverage in India from 1992 to 2006. BMC international health and human rights.

[bib0020] Crocker-Buque T., Mindra G., Duncan R., Mounier-Jack S. (2017). Immunization, urbanization and slums - a systematic review of factors and interventions. BMC public health.

[bib0025] Dempsey A.F., Brewer S.E., Sevick C., Pyrzanowski J., Mazzoni S., O’Leary S.T. (2016). Tdap vaccine attitudes and utilization among pregnant women from a high-risk population. Hum Vaccin Immunother.

[bib0030] Egondi T., Oyolola M., Mutua M.K., Elung’ata P. (2015). Determinants of immunization inequality among urban poor children: evidence from Nairobi’s informal settlements. Int J Equity Health.

[bib0035] Golden S.D., Earp J.A.L. (2012). Social Ecological Approaches to Individuals and Their Contexts:Twenty Years of Health Education & Behavior Health Promotion Interventions. Health Education & Behavior.

[bib0040] Gupta M., Bosma H., Angeli F., Kaur M., Chakrapani V., Rana M. (2017). Impact of a Multi-Strategy Community Intervention to Reduce Maternal and Child Health Inequalities in India: A Qualitative Study in Haryana. PLoS One.

[bib0045] Gupta S.D., Keyl P.M. (1998). Effectiveness of prenatal tetanus toxoid immunization against neonatal tetanus in a rural area in India. The Pediatric infectious disease journal.

[bib0050] Gurnani V., Haldar P., Aggarwal M.K., Das M.K., Chauhan A., Murray J. (2018). Improving vaccination coverage in India: lessons from Intensified Mission Indradhanush, a cross-sectoral systems strengthening strategy. BMJ.

[bib0055] Hu Y., Li Q., Chen Y. (2017). Timeliness of childhood primary immunization and risk factors related with delays: evidence from the 2014 Zhejiang Provincial vaccination coverage survey. Int J Environ Res Public Health.

[bib0060] International Institute for Population Sciences (IIPS) and ICF (2007). National Family Health Survey (NFHS-4), 2005-2006: India.

[bib0065] International Institute for Population Sciences (IIPS) and ICF (2017). National Family Health Survey (NFHS-4), 2015-2016: India.

[bib0070] Jarrett C., Wilson R., O’Leary M., Eckersberger E., Larson H.J. (2015). Strategies for addressing vaccine hesitancy - A systematic review. Vaccine.

[bib0075] Kimman T.G., Boot H.J., Berbers G.A., Vermeer-de Bondt P.E., Ardine de Wit G., de Melker H.E. (2006). Developing a vaccination evaluation model to support evidence-based decision making on national immunization programs. Vaccine.

[bib0080] Lanza S.T., Rhoades B.L. (2013). Latent class analysis: an alternative perspective on subgroup analysis in prevention and treatment. Prev Sci.

[bib0085] MacDonald N.E. (2015). Vaccine hesitancy: definition, scope and determinants. Vaccine.

[bib0090] Maekawa M., Douangmala S., Sakisaka K., Takahashi K., Phathammavong O., Xeuatvongsa A. (2007). Factors affecting routine immunization coverage among children aged 12-59 months in Lao PDR after regional polio eradication in western Pacific region. Biosci Trends.

[bib0095] Ministry of Health & Family Welfare (2014). WHO Certifies India as Polio Free. https://www.mea.gov.in/bilateral-documents.htm?dtl/23154/WHO+Certifies+India+As+Polio+Free.

[bib0100] Ministry of Health and Family Welfare (2008). Immunization Handbook for Medical Officers. http://nihfw.org/pdf/NCHRC-Publications/ImmuniHandbook.pdf.

[bib0105] Nasiru S.G., Aliyu G.G., Gasasira A., Aliyu M.H., Zubair M., Mandawari S.U. (2012). Breaking community barriers to polio vaccination in Northern Nigeria: the impact of a grass roots mobilization campaign (Majigi). Pathog Glob Health.

[bib0110] National Health Mission (2018). Guidelines for Immunization.

[bib0115] Noh J.W., Kim Y.M., Akram N., Yoo K.B., Park J., Cheon J. (2018). Factors affecting complete and timely childhood immunization coverage in Sindh, Pakistan; A secondary analysis of cross-sectional survey data. PLoS One.

[bib0120] Pande R.P., Yazbeck A.S. (2003). What’s in a country average? Wealth, gender, and regional inequalities in immunization in India. Soc Sci Med (1982).

[bib0125] Rots N.Y., Wijmenga-Monsuur A.J., Luytjes W., Kaaijk P., de Graaf T.W., van der Zeijst B.A. (2010). Hepatitis B vaccination strategies tailored to different endemicity levels: some considerations. Vaccine.

[bib0130] Samad L., Tate A.R., Dezateux C., Peckham C., Butler N., Bedford H. (2006). Differences in risk factors for partial and no immunisation in the first year of life: prospective cohort study. BMJ (Clinical research ed).

[bib0135] Travasso C. (2015). Mission Indradhanush makes vaccination progress in India. BMJ (Clinical research ed).

[bib0140] Vikram K., Vanneman R., Desai S. (2012). Linkages between maternal education and childhood immunization in India. Soc Sci Med.

[bib0145] Vojtek I., Dieussaert I., Doherty T.M., Franck V., Hanssens L., Miller J. (2018). Maternal immunization: where are we now and how to move forward?. Ann Med.

[bib0150] World Health Organization (2016). WHO Felicitates India for Yaws, Maternal and Neonatal Tetanus Elimination. https://www.who.int/southeastasia/news/detail/14-07-2016-who-felicitates-india-for-yaws-maternal-and-neonatal-tetanus-elimination.

[bib0155] World Health Organization (2020). Maternal and Neonatal Tetanus Elimination (MNTE). https://www.who.int/immunization/diseases/MNTE_initiative/en/index4.html.

